# Ingredients of Huangqi decoction slow biliary fibrosis progression by inhibiting the activation of the transforming growth factor-beta signaling pathway

**DOI:** 10.1186/1472-6882-12-33

**Published:** 2012-04-03

**Authors:** Jin-Xing Du, Ming-Yu Sun, Guang-Li Du, Feng-Hua Li, Cheng Liu, Yong-Ping Mu, Gao-Feng Chen, Ai-Hua Long, Yan-Qin Bian, Jia Liu, Cheng-Hai Liu, Yi-Yang Hu, Lie-Ming Xu, Ping Liu

**Affiliations:** 1 Key Laboratory of Liver and Kidney Diseases (Ministry of Education), Institute of Liver Diseases, Shuguang Hospital, Shanghai University of Traditional Chinese Medicine, Shanghai 201203, China; 2 E-institute of Shanghai Municipal Education Commission, Shanghai 201203, China; 3 Pharmacy School of Shanghai, University of Traditional Chinese Medicine, Shanghai 201203, China

**Keywords:** Ingredients of Huangqi decoction, Cholestatic liver fibrosis, Transforming growth factor beta 1, Smad-signaling pathway, Extracellular signal-regulated kinase

## Abstract

**Background:**

Huangqi decoction was first described in Prescriptions of the Bureau of Taiping People's Welfare Pharmacy in Song Dynasty (AD 1078), and it is an effective recipe that is usually used to treat consumptive disease, anorexia, and chronic liver diseases. Transforming growth factor beta 1 (TGFβ1) plays a key role in the progression of liver fibrosis, and Huangqi decoction and its ingredients (IHQD) markedly ameliorated hepatic fibrotic lesions induced by ligation of the common bile duct (BDL). However, the mechanism of IHQD on hepatic fibrotic lesions is not yet clear. The purpose of the present study is to elucidate the roles of TGFβ1 activation, Smad-signaling pathway, and extracellular signal-regulated kinase (ERK) in the pathogenesis of biliary fibrosis progression and the antifibrotic mechanism of IHQD.

**Methods:**

A liver fibrosis model was induced by ligation of the common bile duct (BDL) in rats. Sham-operation was performed in control rats. The BDL rats were randomly divided into two groups: the BDL group and the IHQD group. IHQD was administrated intragastrically for 4 weeks. At the end of the fifth week after BDL, animals were sacrificed for sampling of blood serum and liver tissue. The effect of IHQD on the TGFβ1 signaling pathway was evaluated by western blotting and laser confocal microscopy.

**Results:**

Decreased content of hepatic hydroxyproline and improved liver function and histopathology were observed in IHQD rats. Hepatocytes, cholangiocytes, and myofibroblasts in the cholestatic liver injury released TGFβ1, and activated TGFβ1 receptors can accelerate liver fibrosis. IHQD markedly inhibited the protein expression of TGFβ1, TGFβ1 receptors, Smad3, and p-ERK1/2 expression with no change of Smad7 expression.

**Conclusion:**

IHQD exert significant therapeutic effects on BDL-induced fibrosis in rats through inhibition of the activation of TGFβ1-Smad3 and TGFβ1-ERK1/2 signaling pathways.

## Background

Cholestasis, which was identified as an important factor in a variety of chronic liver diseases [1], results in cholestatic liver fibrosis [2]. The main features of cholestatic liver fibrosis that have been implicated include reduction of hepatocytes, proliferation of cholangiocytes, activation of myofibroblasts, and deposition of extracellular matrix (ECM) [3,4].

Transforming growth factor beta 1 (TGFβ1) is a member of the TGFβ superfamily of cytokines, which is known to regulate cell differentiation, proliferation, apoptosis, pro- and anti-inflammatory immune responses, and ECM remodeling [5-7]. Studies have shown that the expression increases of TGFβ1 and TGFβ1 type I receptor (TβRI) is one pathological basis for initiation and development of immunologically-induced fibrosis in bovine serum albumin (BSA) [8]. TGFβ1 elevates ECM synthesis by increasing collagen gene transcription in activated hepatic stellate cells (HSCs) [9]. In addition, evidence has indicated that TGFβ1-mediated bile duct epithelial to mesenchymal transition in hepatic biliary fibrosis [10] and hepatic TGFβ1 activity reduction could inhibit cholestatic fibrosis induced by bile duct ligation (BDL) [11]. Thus, these studies suggest that TGFβ1 is an important factor that is involved in the process of cholestatic liver fibrosis.

TGFβ1 signals through transmembrane Ser-Thr kinase receptors that directly regulate the intracellular Smad pathway [12]. Smads belong to a unique family of signal transduction molecules that can either positively or negatively regulate the transcription of specific genes in response to TGFβ1 signaling [13]. The TGFβ1/Smad signaling pathway plays a prominent role in the activation of HSCs and the regulation of the production, degradation, and accumulation of ECM proteins [14].

Extracellular signal-regulated kinase (ERK) is an important member of the mitogen-activated protein kinase (MAPK) family. Recently, the ERK signal pathway has been found to play an important role in regulating ECM synthesis that was stimulated by TGFβ1 in activated HSCs. Further study has shown that ECM secretion decreased after inhibiting the activation of ERK [15]. Thus, the TGFβ1 signal transduction pathway has become a new effective target for the prevention and treatment of hepatic fibrosis [2,16].

Limited pharmacological therapy for cholestatic liver fibrosis is available, so new therapeutic approaches are expected. Chinese herbal medicine has recently become a hot topic among practitioners of Western medicine. The principles underlying Chinese herbal medicine were established over thousands of years on the basis of clinical experience and practice, while the effective ingredients in most of these medications have not been identified.

Huangqi decoction, also known as Huangqi Liuyi decoction, was first described in Prescriptions of the Bureau of Taiping People's Welfare Pharmacy in the Song Dynasty (AD 1078). It consists of Radix Astragali, Radix Glycyrrhizae, and Fructus Ziziphi Jujubae. Huangqi decoction has been used for treatment of many conditions, including consumptive disease, restlessness, hydrodipsia, anorexia, and chronic liver diseases. The active ingredients were extracted from Huangqi decoction. We have demonstrated previously [15,17] that Huangqi decoction and its ingredients (IHQD) markedly ameliorated hepatic fibrotic lesions that were induced by BDL. In this study, we elucidated the roles of TGFβ1 activation, Smad-signaling pathway, and ERK in the pathogenesis of biliary fibrosis progression and the antifibrotic mechanism of IHQD.

## Methods

### Reagents and antibodies

Methanol, acetonitrile, and water for high-performance liquid chromatography (HPLC) were purchased from Merck (Darmstadt, Germany). Prestained protein marker was purchased from New England Biolabs (Beijing, China). Anti-cytokeratin 7 (CK 7) (sc-25721) rabbit antibody was purchased from Santa Cruz and used in 1:50 dilution. Anti-albumin (Alb) (ab8940) sheep antibody, anti-α-smooth muscle actin (α-SMA) (ab5694) rabbit antibody, and anti-TGFβ1 (ab27969) mouse antibody were purchased from Abcam and prepared in 1:200, 1:200, and 1:1000 dilution, respectively. Anti-TβRI (#3712) rabbit antibody, anti-TβRII (#3713) rabbit antibody, anti-Smad3 (#9513) rabbit antibody, anti-p44/42 MAPK (ERK1/2) (#9107) mouse antibody, and anti-phospho p44/42 MAPK (ERK1/2) (#9106) mouse antibody were purchased from Cell Signaling and diluted 1:1000. Anti-phospho Smad3 (1880-1) rabbit antibody was purchased from Epitomics and used in 1:1000 dilution. Anti-Smad7 (3670-100) rabbit antibody was purchased from BioVision and diluted 1:1000. Secondary fluorescence-labeling goat-anti-mouse Cy3 and goat-anti-rabbit antibody were obtained from Jackson (West Grove, PA, USA) and used in 1:1000 dilution. Labeled rabbit-anti-sheep antibody was obtained from KPL and used in 1:1000 dilution.

### Preparation of IHQD

Huangqi decoction consists of crude herbs in the following dosage: Radix Astragali (30 g), Radix Glycyrrhizae (5 g), and Fructus Ziziphi Jujubae (5 g). The herbal medicines provided by Shanghai Huayu Herbs Co. Ltd. were accredited by a pharmacologist of Shuguang Hospital. The medicinal herb mixture was extracted by boiling water, and the aqueous extracts were vacuum–dried (60°C) to obtain a powder. The aqueous extracts were further extracted by acetic ether and n-butanol, mixed together, purified by a process using MCI gel column chromatography, eluted with 30% methanol, and reclaimed as a dry powder.

### IHQD fingerprinting by high-performance liquid chromatography (HPLC)

A, methanol (HPLC grade, Merck, China); B, water; C, methanol (> 99.99% HPLC grade, Merck, China); seal wash, water/methanol (50/50); needle wash, water/methanol (50/50).

#### Sample solution

One milliliter of the sample was accurately transferred (under content uniformity) into a 50 mL volumetric flask and a suitable volume of methanol was added. Following this, samples were treated with ultrasonic waves for 20 min, placed at room temperature, diluted to volume with 50% methanol, and mixed well.

#### Assay

Five microliters of the sample and standard solution were separately injected into HPLC, the response was recorded (peak area or height), and calculated. First, the column was washed with 100% methanol with 690-740 psi system pressure. The mobile phase was methanol with a flow rate of 1.00 mL/min. Extracted samples were analyzed by HPLC and washed with methanol solution for 30 min in a gradient manner at a flow rate of 1.0 mL/min.

**Table 1:** The methanol gradient elution of the mobile phase

Time (min)	A (%)	B (%)	C (%)
0	0.5	95	4.5
15	2.5	75	22.5
35	4.5	55	40.5
45	8.0	20	72
60	9.5	5.0	85.5

**Figure 1:**
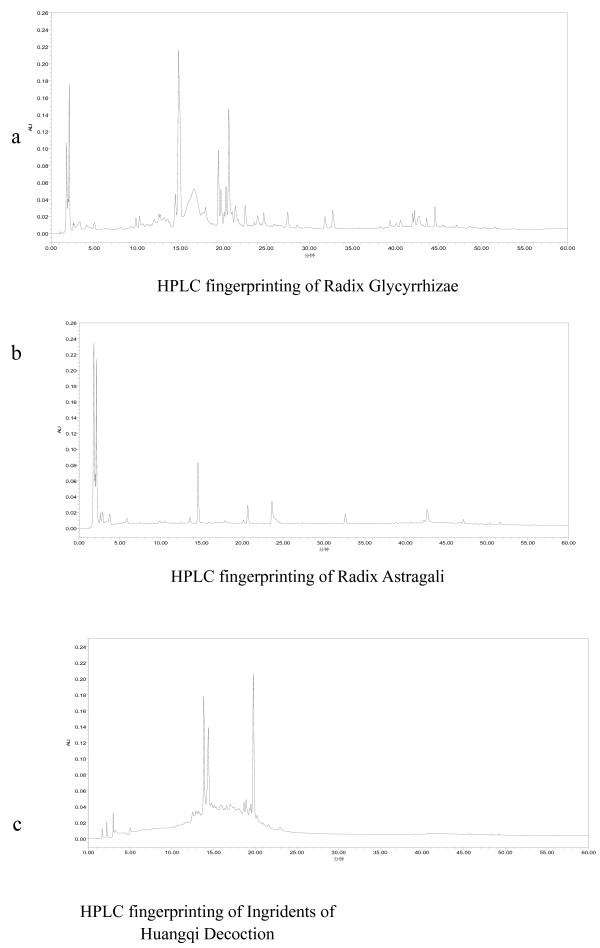
**HPLC fingerprinting of IHQD**. HPLC fingerprinting of Radix Glycyrrhizae (a), Radix Astragali (b), and ingredients of Huangqi decoction (c).

The HPLC fingerprints of the IHQD are illustrated in Figure 1.

### Animals

Male Sprague-Dawley rats (180-220 g) were supplied by the Experimental Animal Center to Chinese Academy of Science. The animals were housed in an air-conditioned room at 25°C with a 12 h dark/light cycle. The rats received humane care with unlimited access to chow food and water during the study. All of this study's protocols were approved by Shanghai University of Traditional Chinese Medicine's Animal Ethics Committee.

### BDL model of liver fibrosis

Rats were anesthetized with pentobarbital and BDL was performed as described previously [16]. Briefly, under sodium pentobarbital anesthesia, the common bile duct was double-ligated with 3-0 silk thread after a midline abdominal incision. The common bile duct was exposed and manipulated but not ligated in the sham control group.

### Grouping of animals and administration

The model rats were randomly divided into 3 experimental groups: sham control group (n = 8), BDL alone group (n = 60) and IHQD group (n = 15). The rats in both sham control group and BDL alone group received water administration (1 mL per 100 g) for 4 weeks, beginning 1 week after operation. The rats in IHQD group received IHQD orally at a concentration of 17.276 mg per 100 g body weight every day for 4 weeks, beginning 1 week after BDL operation.

### Sampling harvesting

Animals in BDL alone group were sacrificed at the end of 1, 2, 3, 4, and 5 weeks after BDL (n = 8-10) for dynamic study, and the rats in both sham control group and IHQD group were sacrificed at the fifth weekend after surgery by pentobarbital anesthesia (50 mg/kg, intraperitoneal injection). Blood samples were obtained from interior vena cava, centrifuged at 3000 r/min for 30 min at 4°C after 3 h, and serum was kept at -70°C for liver function tests. The livers were rapidly removed, and liver tissue was taken from the right lobe of the liver, fixed in 10% phosphate-buffered formaldehyde, routinely processed, and blocked into paraffin for detection of collagen content by biochemical methods and image analysis. Some liver tissues were immersed immediately into cyromatrix (Tissue-Tek OCT, Sakura Finetek, Torrance, CA, USA), while other liver tissue was snap-frozen in liquid nitrogen and stored at -70°C.

### Histological examination

#### Hematoxylin and eosin (H&E) stain

Formalin-fixed liver tissues were processed, and 4 μm thick paraffin sections were stained with H&E for 10 min, rinsed with water, and then placed in 75% HCl-ethanol for 30 s, rinsed with water, placed in eosin-ethanol for 1-2 min, dehydrated, and mounted.

#### Sirius red stain

Formalin-fixed liver tissues were processed, and 4 μm thick paraffin sections were stained with Sirius red for 60 min, rinsed with 100% ethanol, dehydrated, and mounted.

### Hydroxyproline content in liver tissue and serum biochemistry examination

Liver tissue (100 mg) was prepared for hydroxyproline (Hyp) determination according to a modified method by Jamall et al. [18]. Briefly, liver samples were homogenized and hydrolyzed in 6 N HCl at 110°C for 18 h. After filtration of the hydrolysate through a 0.45 mm Millipore filter, chloramine-T was added to a final concentration of 2.5 mM. The mixture was then treated with 410 mM paradigm ethyl-amino-benzaldehyde and incubated at 60°C for 30 min. After cooling to room temperature, the absorbance of samples was read at 560 nm against a reagent blank that contained all reagents except tissue. The Hyp content in each sample was determined from a standard and was expressed as micrograms per gram of wet weight (μg/g).

Serum levels of albumin (Alb), alanine aminotransferase (ALT), and total bilirubin (TBil) were measured following the instructions provided by the manufacturer.

### Laser confocal microscopy

Liver samples were immersed into cyromatrix and cut into 5 μm thick cryosections. The cryosections were kept at room temperature for 10 min, placed in 100% acetone for 10 min, and washed 3 times with PBS. Thereafter, the cryosections were incubated with 5% bovine serum albumin (BSA) at 37°C for 30 min and incubated with monoclonal anti-TGFβ1 primary antibody at 37°C for 2.5 h. Slides were then washed 3 times with PBS and incubated with the secondary Cy3-conjugated affinipure goat anti-mouse antibody at 37°C for 1 h. After being washed, the sections were incubated with α-SMA, Alb, or CK 7 primary antibody at 37°C for 2.5 h, incubated with the secondary goat anti-rabbit, rabbit anti-sheep, or goat anti-rabbit antibody, respectively, at 37°C for 1 h, and covered with mounting medium. Imaging analyses were performed using a Leica (Mannheim, Germany) DMIRBE inverted stand and Leica TCS2MP confocal system. A computer-aided morphometric analysis was used to quantitatively determine the positive staining area. Five fields of view were selected randomly from every slice, and the even integration optic density (IOD) value of the positive staining area was corrected by the blank in the same visual field.

### Immunoblot analysis

Liver samples were lysed by RIPA buffer containing 50 mM Tris-HCl (pH 7.2), 150 mM NaCl, 1% NP-40, 0.1% SDS, 1 mM EDTA, and 1 mM PMSF and homogenized in ice-water for 3 times for 10 s at 10,000 rpm. The homogenized solution was transferred to a 1.5 mL Oak Ridge tube containing 0.1 M PMSF (0.1 mL), centrifuged for 10 min at 4°C and 12,000 rpm to obtain supernatant. Then, the total protein concentration was determined by Bio-Rad Dc protein Assay Reagent. Proteins were electrophoretically resolved by 10% SDS polyacrylamide gel and immobilized on Hybond-ECL nitrocellulose membranes. TRIS buffered saline (20 mM TRIS, 150 mM NaCl, pH 7.4) with 0.1% Tween-20 and 5% non-fat dairy milk was used to block non-specific binding sites. Blots were incubated with primary antibodies at 4°C overnight and with secondary antibodies at room temperature for 1 h. Bound antibodies were visualized using an enhanced chemiluminescence kit (Pierce, Rockford, IL, USA) and exposed to Kodak BioMax film (Kodak, Rochester, NY, USA).

### Statistical analysis

All data were presented as mean ± standard deviation. Statistical testing was performed with SPSS software version 12.0. Comparison between groups was performed with test of homogeneity of variances and one-way analysis of variance (ANOVA), and post hoc analysis was performed using Bonferroni or Dunnett's multiple comparison test. Correlation coefficients were calculated by Spearman's rank correlation method. *P*-values less than 0.05 were considered statistically significant.

## Results

### Histological changes

**Figure 2:**
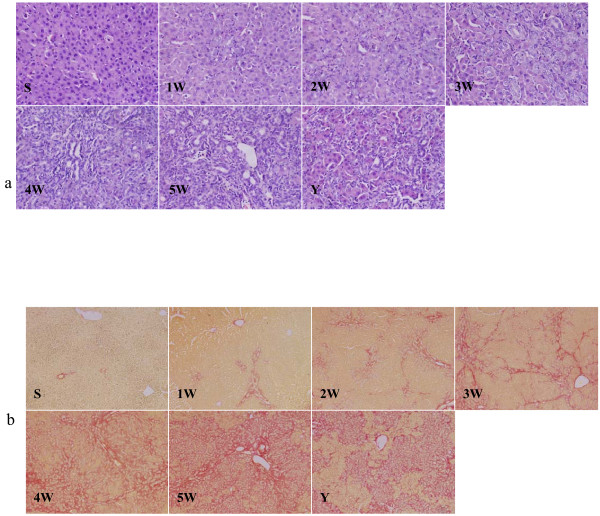
**Liver tissue H&E Staining (× 400) and Sirius red staining (× 100)**. S, sham control group; 1 W-5 W, one to five weeks BDL alone groups; Y, IHQD group.

H&E stain showed that no morphological abnormality was observed in sham control rats. Persistent reduction of hepatocytes, gradual proliferation of cholangiocytes, and continuous infiltration of neutrophils were noted in BDL alone rats. Moreover, hepatocytes aggregate to flower cricoids at 2 weeks, and tubular structures were observed at 3, 4, and 5 weeks after BDL. Compared to these observations that were made in rats in BDL alone group, there were obvious improvements in IHQD group (Figure 2a).

Sirius red stain showed that collagen staining was scarcely observed in sham control liver samples except in the area around small central venous walls. In BDL alone rats, extensive peribiliary and interstitial collagen deposits were evident. However, IHQD markedly inhibited hepatic collagen accumulation after BDL (Figure 2b).

### Changes in serum liver function and liver tissue Hyp content

Serum ALT activity, TBil, and liver tissue Hyp content increased gradually, and peaked at the end of the fifth week, while Alb content decreased gradually and reached its lowest level at the end of the fifth week in BDL alone group compared to values observed in sham control group (*p *< 0.01). Compared to measurements in the 5th week BDL alone group, serum ALT activity, contents of serum TBil, and liver tissue Hyp significantly declined (*p *< 0.05, *p *< 0.01, *p *< 0.01) and serum Alb content remarkably increased in IHQD group (*p *< 0.05) (Table 2).

**Table 2:** Changes of serum liver function and liver tissue hydroxyproline content in each group (mean S.D.)

group	n	TBil(mg/dl)	Alb(g/l)	ALT(u/L)	Hyp (μg/g wet liver)
sham control group	8	0.90 ± 0.28##	25.62 ± 1.98##	12.92 ± 1.13##	164.90 ± 12.41##

1 week BDL alone group	8	5.24 ± 0.85**#	23.10 ± 2.45##	69.18 ± 2.82**##	292.96 ± 93.68**##

2 week BDL alone group	8	5.51 ± 0.80**#	20.97 ± 0.39**##	31.68 ± 4.00**##	326.93 ± 14.45**##

3 week BDL alone group	8	5.58 ± 0.27**	20.12 ± 1.57**##	38.96 ± 8.15**##	804.70 ± 35.50**##

4 week BDL alone group	8	6.16 ± 0.95**	16.4 ± 3.15**##	56.83 ± 9.96**	808.37 ± 65.93**##

5 week BDL alone group	10	6.72 ± 0.24**	12.48 ± 3.22**	56.38 ± 5.79**	1069.03 ± 69.27**

IHQD group	14	3.08 ± 0.60**##	15.18 ± 2.99**#	48.81 ± 3.57**#	853.85 ± 21.4**##

Note: Compared with sham control group, *p < 0.05, **p < 0.01; compared with 5 week BDL alone group, #*p *< 0.05, ##*p *< 0.01. *TBil *total bilirubin, *ALT *alanine aminotransferase, *Alb *albumin, *Hyp *hydroxyproline

### Changes in protein expression of TGFβ1, α-SMA, Alb, and CK 7 in liver tissue

**Figure 3:**
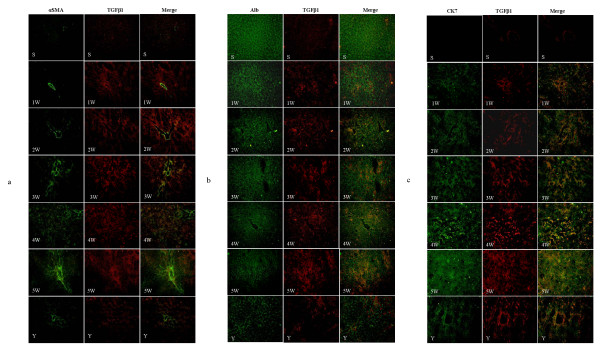
**Changes in TGFβ1, α-SMA, Alb, and CK 7 protein expression detected using confocal microscopy (× 200)**. S, sham control group; 1 W-5 W, one to five weeks BDL alone groups; Y, IHQD group (a) TGFβ1 (red), α-SMA (green), merge (yellow); (b) TGFβ1 (red), Alb (green), merge (yellow); (c) TGFβ1 (red), CK 7 (green), merge (yellow).

As shown in Figure 3a, the myofibroblast marker α-SMA (green) was seen only in the hepatic central vein wall in the portal triads of sham control liver, while TGFβ1 (red) staining and double staining for both markers (yellow) were light. In BDL alone group, positive staining for α-SMA, TGFβ1, and double staining increased gradually with modeling. α-SMA positive staining was predominantly localized in the portal triads at 1, 2, and 3 weeks after BDL. However, strong staining for α-SMA was observed around proliferated cholangiocytes at 4 and 5 weeks after BDL. Compared with 5 week BDL alone group, positive staining for α-SMA, TGFβ1, and double staining was significantly decreased in IHQD group (*p *< 0.01).

Strong staining for the hepatocyte marker Alb (green) and faint staining for TGFβ1 (red) were observed, but positive double staining for both markers (yellow) was completely negative in sham control group (Figure 3b). With progress of modeling, positive staining for Alb decreased gradually; however, positive staining for TGFβ1 and positive double staining increased gradually. Compared with 5 week BDL alone group, positive staining for Alb increased significantly, but positive staining for TGFβ1 and positive double staining for them decreased significantly in IHQD group (*p *< 0.01).

As shown in Figure 3c, staining for the cholangiocyte marker CK 7 (green) was completely negative, and TGFβ1 staining (red) and double staining for both markers (yellow) were light in sham control group. With progress of modeling, positive staining for CK 7, TGFβ1, and positive double staining increased gradually, especially at 4 and 5 weeks after BDL. Compared with 5 week BDL alone group, positive staining for CK 7, TGFβ1, and positive double staining was significantly decreased in IHQD group (*p *< 0.01).

### Correlation of TGFβ1 with α-SMA, Alb, and CK 7

**Figure 4:**
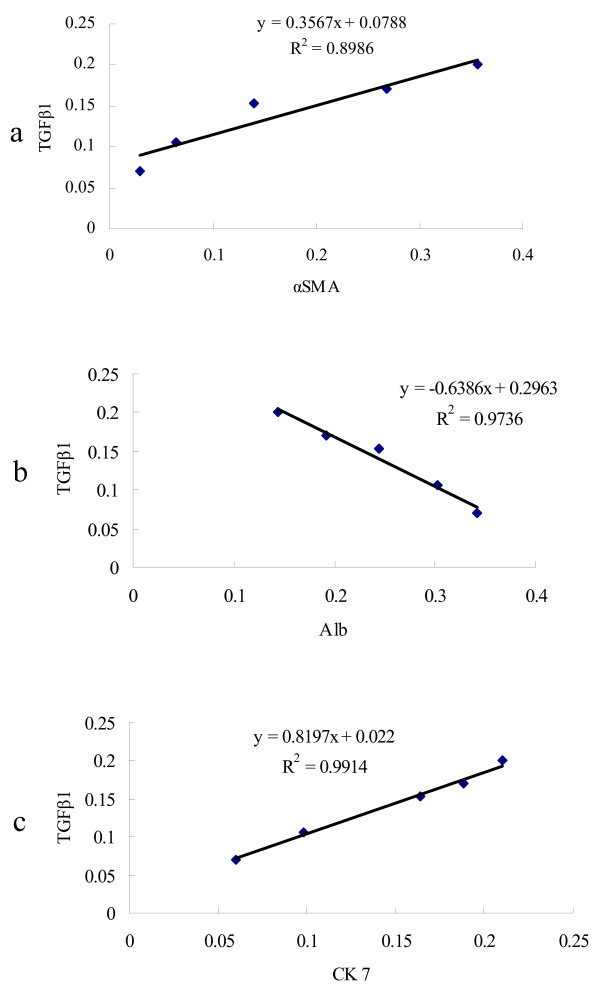
**Correlation of positive staining area of TGFβ1 with α-SMA, Alb, and CK 7 protein expression in 1 to 5 weeks BDL alone group**. (a) Correlation analysis between TGFβ1 and α-SMA, (b) correlation analysis between TGFβ1 and Alb, (c) correlation analysis between TGFβ1 and CK 7.

The IOD values of TGFβ1, α-SMA, and CK 7 positive staining areas were calculated (Table 3) and the relationships between TGFβ1 and α-SMA, TGFβ1 and Alb, and TGFβ1 and CK 7 were analyzed. The IOD value of the TGFβ1 positive staining area was positively correlated with the level of α-SMA (r_s _= 0.8984, *p *< 0.01, Figure 4a) and CK 7 (r_s _= 0.8572, *p *< 0.01, Figure 4c), and was negatively correlated with the level of Alb (r = -0.8694, *p *< 0.01, Figure 4b) in BDL alone group.

**Table 3:** The average integration optic density (IOD) values of positive staining areas of α-SMA, Alb, CK 7, and TGFβ1 in liver tissues (mean ± S.D.)

group	n	α-SMA	Alb	CK 7	TGFβ1
sham control group	8	0.02 ± 0.01##	0.53 ± 0.03##	0.00 ± 0.00##	0.03 ± 0.01##

1 week BDL alone group	8	0.03 ± 0.01##	0.34 ± 0.02**##	0.06 ± 0.02**##	0.07 ± 0.02*##

2 week BDL alone group	8	0.06 ± 0.01**##	0.30 ± 0.02**##	0.10 ± 0.02**##	0.11 ± 0.03**##

3 week BDL alone group	8	0.14 ± 0.02**##	0.24 ± 0.02**##	0.16 ± 0.02**##	0.15 ± 0.03**##

4 week BDL alone group	8	0.27 ± 0.03**##	0.19 ± 0.02**##	0.19 ± 0.01**	0.17 ± 0.02**

5 week BDL alone group	10	0.36 ± 0.03**	0.14 ± 0.02**	0.21 ± 0.02**	0.20 ± 0.02**

IHQD group	14	0.11 ± 0.02**##	0.28 ± 0.01**##	0.15 ± 0.02**##	0.15 ± 0.03**##

Note: Compared with sham control group, *p < 0.05, **p < 0.01; compared with 5 week BDL alone group, #*p *< 0.05, ##*p *< 0.01. *α-SMA *α-smooth muscle actin, *Alb *albumin, *CK 7 *cytokeratin 7, *TGFβ1 *transforming growth factor β1

3.5 Changes in the protein expression of α-SMA, TGFβ1, TβRI, TβRII, Smad3, phospho-Smad3, Smad7, ERK1/2, and phospho-ERK1/2 in liver tissue

**Figure 5:**
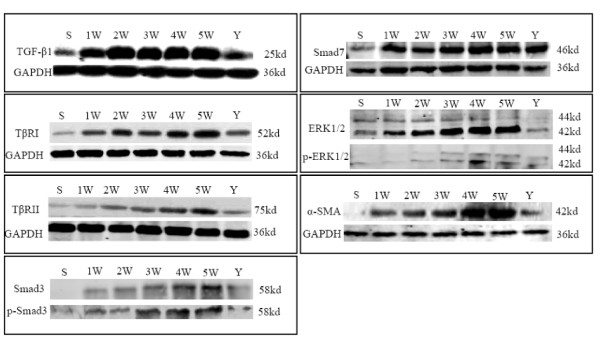
**Changes in TGFβ1, TβRI, TβRII, Smad3, phospho-Smad3, ERK1/2, phospho-ERK1/2, Smad7, and α-SMA protein expression detected by western blotting**. S, sham control group; 1 W-5 W, one to five weeks BDL alone groups; Y, IHQD group.

We observed low levels of protein expression of TGFβ1, TβRI, TβRII, ERK1/2, phospho-ERK1/2, and Smad7 in sham control liver tissue, and no obvious protein expression of Smad3, phospho-Smad3, and α-SMA. Compared to the protein expression levels in sham control group rats, protein expression of α-SMA, TβRI, TβRII, Smad3, phospho-Smad3, ERK1/2, and phospho-ERK1/2 markedly increased with progress of modeling in BDL alone group; these increases were especially evident at 4 and 5 weeks after BDL. However, there were no distinct changes in TGFβ1 and Smad7 protein expression from 1 to 5 weeks in BDL alone group. The levels of protein expression of all proteins discussed above except Smad7 were significantly decreased in IHQD compared to BDL alone (Figure 5).

## Discussion

BDL is an established animal model of cholestasis with complete biliary obstruction and accumulation of multiple primary bile acids in liver and serum [19]. The changes that are observed in the BDL model of cholestasis parallel those that occur in cholestasis in humans [20]. A previous study has indicated that TGFβ1 plays a prominent role in stimulating liver fibrogenesis through myofibroblasts that are derived from HSCs [10] during the development of chronic liver injury, including inflammation, fibrosis, and regeneration. In this study, IHQD significantly ameliorated the cholestatic liver fibrosis in BDL rats, and IHQD inhibited the protein expression of TGFβ1.

TGFβ1, a prototype of multifunctional cytokines, has been proposed to be a "master switch" in the induction of fibrosis [21]. Upregulation of TGFβ1 expression is a consistent feature of most fibrotic disease [22-24]. Cells, such as HSCs, Kupffer cells, myofibroblasts, endothelial cells, and invading mononuclear cells, could synthesize and release TGFβ1 [12]. Many studies have created a strong rationale for an antifibrotic strategy in which the principal objective of treatment is blocking TGFβ1.

Our work suggested that hepatocytes, cholangiocytes, and myofibroblasts could synthesize and release TGFβ1. In return, TGFβ1 promoted reduction of hepatocytes, proliferation of cholangiocytes, and activation of myofibroblasts (Figures 2, 3, 5). IHQD suppressed TGFβ1 protein expression and thus suppressed the effects that TGFβ1 protein expression had on these cell types. These results suggested that TGFβ1 was a plausible candidate that may play a central role in mediating cholestatic fibrosis, and that suppression of TGFβ1 expression may be an effective target of IHQD antifibrotic injury.

TGFβ1 signaling occurs through heteromeric complexes of type I and type II receptors (TβRI, TβRII) and cytoplasmic protein mediators belonging to the Smad family [25]. The activation of the receptor complexes occurs when TβRII transphosphorylates the GS domain of TβRI. The activated TβRI associates transiently with, and also phosphorylates, the receptor-regulated Smads (R-Smads), Smad2 and Smad3. Once phosphorylated, R-Smads dissociate from the receptor, bind to Smad4, and enter the nucleus. The activated Smad complex binds to target promoters in association with DNA-binding cofactors, and recruits coactivators to activate transcription. Alternatively, activated Smad complex can also recruit corepressors, which in turn bind histone deacetylases. As a result, Smads can either positively or negatively regulate the transcription of specific genes in response to TGFβ1 signaling [25-27]. Antagonistic Smad7 competes with R-Smads for binding sites to activate TβRI and thus prevents the phosphorylation of R-Smads, resulting in receptor degradation [28]. Therefore, Smad7 terminates or reduces the strength of the TGFβ1-R-Smads signal in a negative feedback loop.

In this study, focusing on the roles of Smad3 and antagonistic Smad7, we investigated the differential regulatory mechanisms of the TGFβ1 signal in rats during chronic cholestatic liver injury. Our data suggested that in the cholestatic liver injury, hepatocytes, cholangiocytes, and myofibroblasts released TGFβ1 and activated TGFβ1 receptors. TGFβ1 bound its receptor, phosphorylated Smad3, and accelerated liver fibrosis. A remarkable finding noted in other data was that cholestasis induced TGFβ signaling via Smad3 in vivo [29]. In liver wound healing, Smad3 is required for hepatic stellate cell matrix production and matrix interactions [9,30], as well as maximal type I collagen induction [31]. In addition, high-level expression of Smad7 protein was also observed. In the process of cholestatic liver fibrosis, Smad7 antagonized Smad3-mediated TGFβ1 signal in a negative feedback loop without interfering with fibrogenesis. This finding is consistent with previous work [29]. IHQD markedly inhibited the protein expression of Smad3 without changing Smad7 expression, suggesting that IHQD ameliorated cholestatic liver fibrosis by preventing Smad3-mediated TGFβ1 signal in a positive feedback loop.

Recent findings have suggested a more complex paradigm of TGFβ1 signaling wherein Smads interact with other signaling cascades, including the MAPK pathway [32]. ERK is an important member of the MAPK family. A study demonstrated that TGFβ signaling that was activated after BDL was mediated through ERK activation. The decrease of TGFβ1-induced transcriptional activity by the ERK blockade results from direct suppression of R-Smad dependent transcriptional activation. In addition, differential inhibition of phosphorylation at different Smad serines suggests mechanisms of crosstalk between the Smad and MAPK pathways, which would account for partial inhibition of TGFβ/Smad signaling by MAPK pathway inhibitors [33]. These results strongly suggest a synergizing role for ERK signal in Smad-signaling that is initiated by TGFβ1.

In this study, we found that ERK1/2 was activated after BDL, and crosstalk between ERK1/2 and the Smad-signaling pathway enhanced TGFβ1-dependent responses in cholestatic fibrosis caused by BDL. IHQD inhibited ERK1/2 activation after BDL. IHQD may exert its suppressive effects on cholestatic liver fibrosis, at least in part, through suppression of ERK1/2 activation and crosstalk between ERK1/2 and the Smad-signaling pathway.

The multifunctional characteristics of TGFβ1 indicate a need for tight control of its signaling. Indeed, both positive and negative regulatory mechanisms have been observed at nearly every step in the TGFβ1 signaling cascade, from release of biologically active ligands to the Smad-mediated transcriptional effects [34]. Our results demonstrated that TGFβ1-mediated induction of Smad3, Smad7, and ERK1/2 was involved in this tight regulation of its fibrosis signals in cholestatic liver fibrosis. Smad7 expression increased but did not interfere with fibrogenesis. The TGFβ1 signal through phosphorylation of Smad3 and activation of ERK1/2 was constantly propagated throughout hepatic biliary injury.

Understanding the differential regulatory mechanisms of the TGFβ1 signal between physiologic and pathologic situations will be essential in the design of new therapeutic approaches for various diseases caused by a deregulation of the TGFβ1 signal. Thus, antagonists of the TGFβ1 signal could be applied in cholestatic liver fibrosis. Our findings suggested that IHQD suppressed the cholestatic liver fibrosis by inhibiting TGFβ1 signal-mediated activation of Smad3 and ERK1/2. This provides scientific evidence for the clinical application of Huangqi decoction in treatment of cholestatic liver fibrosis.

## Conclusions

Our study indicated that IHQD exerted a significant therapeutic effect on BDL-induced fibrosis in rats by inhibiting the activation of TGFβ1-Smad3 and TGFβ1-ERK1/2 signaling pathways. These findings provide scientific evidence for the clinical application of Huangqi decoction in treatment of cholestatic liver fibrosis.

## Competing interests

The authors declare that they have no competing interests.

## Authors' contributions

JXD and MYS carried out the study and designed the experiments. GLD, FHL, CL, YPM, and YQB contributed reagents, materials, and analysis tools. GFC, AHL, and JL contributed BDL model. CHL, YYH, and LMX analyzed data. MYS and GLD supervised work and corrected the manuscript. PL conceived and designed experiments and wrote the manuscript. All authors read and approved the final manuscript.

## Pre-publication history

The pre-publication history for this paper can be accessed here:

http://www.biomedcentral.com/1472-6882/12/33/prepub
